# Anti-*Candida* Activity of Cysteine-Modified Amidated Decoralin in the Presence of Engineered Nanomaterials

**DOI:** 10.3390/pharmaceutics17040460

**Published:** 2025-04-02

**Authors:** Vânia Rocha, Helena Almeida, Bruno Sarmento, José das Neves

**Affiliations:** 1 i3S–Instituto de Investigação e Inovação em Saúde, Universidade do Porto, Rua Alfredo Allen 208, 4200-135 Porto, Portugal; vrocha@i3s.up.pt (V.R.); helena.almeida@i3s.up.pt (H.A.); bruno.sarmento@i3s.up.pt (B.S.); 2 ICBAS–Instituto de Ciências Biomédicas Abel Salazar, Universidade do Porto, Rua de Jorge Viterbo Ferreira 228, 4050-313 Porto, Portugal; 3 Instituto Universitário de Ciências da Saúde, CESPU, Rua Central de Gandra 1317, 4585-116 Gandra, Portugal

**Keywords:** antifungal agents, antimicrobial peptides, candidiasis, drug resistance, nanomedicine

## Abstract

**Background:** Candidiasis remains a chief concern in global healthcare. Drug safety issues and increasing resistance make it urgent to develop alternative antifungal agents, namely antimicrobial peptides. Amidated decoralin (Dec-CONH_2_) possesses considerable anti-*Candida* activity, and its association with nanocarriers could help in enhancing efficacy while reducing intrinsic toxicity to the host. **Methods:** We studied an *N*-terminal cysteine-modified version of the peptide (Cys-Dec-CONH_2_) and screened the effects of different nanosystems (polymeric nanoparticles (NPs), liposomes and gold NPs) on its activity against azole-sensitive and azole-resistant *Candida* species using a clinically relevant in vitro assay. **Results:** The antifungal activity of Cys-Dec-CONH_2_ was maintained (minimum inhibitory concentration (MIC) = 16–64 µg/mL), but the presence of poly(d,l-lactic-co-glycolic acid) (PLGA)- and polycaprolactone-based NPs impaired the antifungal effect of the peptide (MIC > 256 µg/mL). This effect was milder for polystyrene-based NPs, liposomes, and gold NPs (MIC ≤ 128 µg/mL). Additionally, the covalent surface functionalization of PLGA-based NPs with Cys-Dec-CONH_2_ or the presence of relevant biomolecules (albumin and mucin) resulted in complete inhibition of antifungal activity. **Conclusions:** Our data suggest that Cys-Dec-CONH_2_ is able to establish strong interfacial interactions with different nanomaterials, which need to be considered when developing nanomedicines based on this peptide for the management of candidiasis.

## 1. Introduction

Candidiasis includes all infections caused by fungi of the genus *Candida*. These can be generally divided into superficial candidiasis, which is typically mild and affects the skin or mucosal surfaces, or invasive (systemic) infections, which—despite being less common—are associated with greater morbidity and can lead to death [1]. *Candida* spp. are typically commensal yeasts in humans, being able to become opportunistic pathogens in various circumstances [2]. Onset of pathogenicity on the skin and mucosae involves adhesion to epithelia followed by tissue colonization. Some species are able to undergo dimorphic transition into hyphal form, which promotes epithelial invasion and causes deeper infection. This usually triggers an effective local host immune response, while, on some occasions, *Candida* can still escape and lead to colonization of the vascular system and candidemia. Invasive candidiasis can also be triggered by direct blood contact, namely in healthcare facilities [3].

*C. albicans* is the main pathogen involved in candidiasis, but the prevalence of other non-albicans species such as *C. glabrata*, *C. tropicalis*, and *C. krusei*, among others, is increasing. Current treatment options involve the use of several classes of antifungals, with azoles being considered as first choice [4,5]. Drugs from this class hinder ergosterol synthesis by inhibiting lanosterol 14-alpha-demethylase, thus impairing fungal cell membrane integrity. Others such as polyenes (binds to ergosterol and destabilizes the cell membrane), echinocandins (targets the synthase responsible for β-(1,3)-D-glucan production, thus disrupting the cell wall) and fluoropyrimidines (interferes with fungal RNA and protein synthesis after activation by cytosine deaminase) are of increasing importance, namely in cases of systemic infection or azole resistance [6,7]. However, there are several limitations regarding pharmacokinetics, drug interactions, and side effects of clinically available drugs that narrow their therapeutic efficacy and safety [5]. Moreover, the increase in the number of resistant cases of candidiasis is a major public health concern, which, by itself, justifies the need for developing alternative therapeutics [8,9].

The search for new antifungals has been ongoing, but only a few novel drugs have been introduced over the last decade. Honorable mentions include ibrexafungerp and oteseconazole (oral triterpenoid and oral azole, approved in 2021 and 2022 by the FDA, respectively, for managing vulvovaginal candidiasis) [10,11], and rezafungin (an intravenous echinocandin approved in 2023 by the FDA for the treatment of invasive candidiasis) [12]. Despite these additions, the currently available armamentarium would benefit tremendously from the development of antifungals with new mechanisms of action [13]. Antimicrobial peptides have generated considerable enthusiasm in the scientific community as an alternative drug class for fighting different types of infection [14]. In particular, these relatively small amino acid sequences seem to be promising candidates for managing candidiasis, presenting broad in vitro anti-*Candida* activity [15,16,17,18,19] and mechanisms of action different from those of approved antifungals (e.g., pore formation in the cell wall, membrane disruption, mitochondrial damage, induction of reactive oxygen species generation, DNA damage) [20]. One particularly promising molecule is decoralin (Dec), a linear cationic α-helical peptide containing 11 amino acids (Ser-Leu-Leu-Ser-Leu-Ile-Arg-Lys-Leu-Ile-Thr) that was first extracted from the venom of the potter wasp *Oreumenes decorates* [21]. It has been shown to possess broad-spectrum antimicrobial activity (including against *C. albicans*), which is enhanced upon amidation of the *C*-terminal (Dec-CONH_2_) [21,22]. The short size and linear structure of Dec and Dec-CONH_2_ facilitate chemical modification and conjugation with other moieties that could help in further enhancing its antimicrobial activity while reducing toxicity to mammal cells [23,24,25]. In particular, the combination of the peptide with a nanocarrier could provide an interesting strategy for enhancing delivery and ultimately the activity against *Candida* species. Such an approach has been successfully tested for other antimicrobial peptides. For example, loading octominin (Gly-Trp-Leu-Ile-Arg-Gly-Ala-Ile-His-Ala-Gly-Lys-Ala-Ile-His-Gly-Leu-Ile-His-Arg-Arg-Arg-His) into chitosan nanoparticles (NPs) enhanced its activity against *C. albicans* as compared to the free peptide [26]. Similarly, conjugation of indolicidin (Ile-Leu-Pro-Trp-Lys-Trp-Pro-Trp-Trp-Pro-Trp-Arg-Arg) to the surface of gold NPs (Au NPs) was shown to be useful in increasing roughly four- to six-fold the antifungal activity against azole-resistant clinical isolates of *C. albicans* [27].

The purpose of this work was to screen different engineered nanomaterials (polymeric NPs, gold NPs, and liposomes) for suitability as potential carriers of Dec-CONH_2_ modified with a cysteine on its *N*-terminal (Cys-Dec-CONH_2_). The addition of this amino acid containing a thiol group was conducted to allow specific terminal covalent bonding of the peptide to maleimide-modified molecules. In particular, we tested the effect of the presence of nanomaterials on the anti-*Candida* effect of Cys-Dec-CONH_2_, including upon physical or chemical association.

## 2. Materials and Methods

### 2.1. Materials

Dec-CONH_2_ (Ser-Leu-Leu-Ser-Leu-Ile-Arg-Lys-Leu-Ile-Thr-CONH_2_) and its *N*-terminal (Cys-Dec-CONH_2_; Cys-Ser-Leu-Leu-Ser-Leu-Ile-Arg-Lys-Leu-Ile-Thr-CONH_2_) and *C*-terminal (Dec-CONH_2_-Cys; Ser-Leu-Leu-Ser-Leu-Ile-Arg-Lys-Leu-Ile-(Thr-CONH_2_)-Cys) cysteine-modified derivatives were obtained from Pepmic (Suzhou, China). Carboxyl-terminated poly(d,l-lactic-co-glycolic acid) (PLGA; Purasorb PDLG 5002A, 50:50 d,l-lactide:glycolide, MW = 17 kDa) was kindly provided by Corbion (Amsterdam, The Netherlands). PLGA-poly(ethylene glycol)-maleimide (PLGA-PEG-Mal; MW = 30:5 kDa) and 2-distearoyl-*sn*-glycero-3-phosphoethanolamine-PEG-Mal (DSPE-PEG-Mal; PEG MW = 10 kDa) were obtained from Ruixibiotech (Xi’an City, China); PLGA-PEG (MW ≈ 45:5 kDa) from Polyscitech (West Lafayette, IN, USA); polycaprolactone (PCL; MW ≈ 14 kDa), cholesterol, 3-morpholinopropane-1-sulfonic acid (MOPS), tris(2-carboxyethyl)phosphine (TCEP), albumin from human serum, mucin type II from porcine stomach, and Roswell Park Memorial Institute (RPMI) 1640 medium from Sigma-Aldrich (Saint Louis, MO, USA); poloxamer 407 from BASF (Ludwigshafen, Germany); Lipoid P 45 (lecithin fraction with 45% phosphatidylcholine) from Lipoid (Ludwigshafen, Germany); Fluconazole, clotrimazole, 200 nm carboxylate modified polystyrene NPs (PS NPs), Sabouraud dextrose agar (SDA), and McCoy’s 5A medium from Thermo Fisher Scientific (Waltham, MA, USA); heat-inactivated fetal bovine serum and penicillin/streptomycin from Biochrom GmbH (Berlin, Germany); and 50 nm Au NPs from Applied Nanoparticles (Barcelona, Spain). All other materials, reagents, and solvents were of analytical grade or equivalent.

### 2.2. Production of Nanosystems

**Figure 1:**
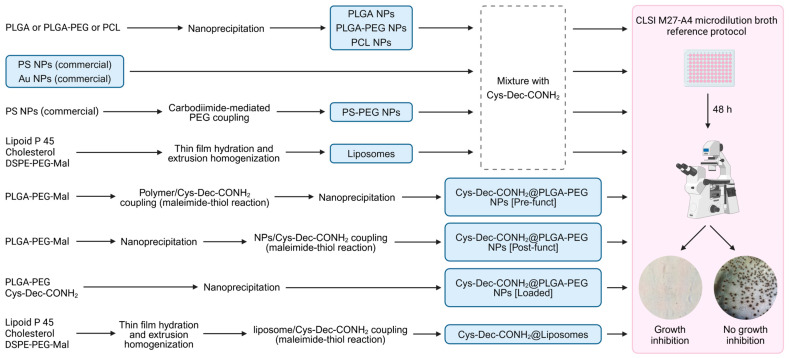
Schematic representation of the general methodology to obtain nanosystems (highlighted in blue boxes) and assess antifungal activity of Cys-Dec-CONH_2_. Representative microscopy images for *Candida* spp. growth and no growth inhibition are also included (40× magnification).

Different nanosystems were obtained as schematically shown in Figure 1. PLGA NPs and PCL NPs were produced by nanoprecipitation using protocols previously reported [28,29,30]. In brief, 20 mg of polymer was dissolved in 2 mL of acetone and injected into 10 mL of 1% (*w*/*v*) poloxamer 407 under stirring (300 rpm) at room temperature. After 2 h under stirring, NPs were washed twice with ultrapure water using centrifugal filters (Amicon Ultra-15, 100 kDa MWCO, Merck Millipore, Burlington, MA, USA) at 1500× *g* for 10 min to remove excess stabilizer. Finally, the colloidal dispersion volume was adjusted to 1 mL with ultrapure water and stored at 4 °C until further use. PLGA-PEG NPs and PLGA-PEG-Mal NPs were also prepared similarly by replacing 4 mL of PLGA with the respective co-polymer.

Large unilamellar liposomes were prepared by the tandem thin-film hydration method and extrusion homogenization [31,32]. Briefly, Lipoid P 45 (28.8 mg), cholesterol (3.2 mg), and DSPE-PEG-Mal (8 mg) were dissolved in 5 mL of chloroform and placed in a round-bottom flask. The organic solvent was evaporated under argon atmosphere at room temperature, and the obtained dry lipid film rehydrated with 4 mL of ultrapure water at approx. 75 °C (to achieve a final concentration of 10 mg/mL) and vortexed [33,34]. The vesicle dispersion was then homogenized by extrusion (3–4 cycles) using a polycarbonate membrane (0.2 μm pore size) mounted on a micro extruder (Avanti Polar Lipids, Alabaster, AL, USA) maintained at 75 °C. Finally, the extruded large unilamellar vesicles were concentrated to a final volume of 1 mL using Amicon Ultra-15 centrifugal filters (1500× *g* for 1 h at 20 °C). Samples were stored at 4 °C until use.

### 2.3. Association of Cys-Dec-CONH_2_ with Nanosystems

Cys-Dec-CONH_2_ was covalently linked to PLGA-PEG-Mal NPs or liposomes containing DSPE-PEG-Mal via maleimide-thiol reaction. In the case of PLGA-PEG-Mal NPs, two distinct approaches were used, namely (i) by conjugating the reactive co-polymer to Cys-Dec-CONH_2_ before the production of NPs (Cys-Dec-CONH_2_@PLGA-PEG NPs [Pre-funct]) or (ii) by surface functionalization of pre-prepared PLGA-PEG-Mal NPs with the peptide (Cys-Dec-CONH_2_@PLGA-PEG NPs [Post-funct]). Liposomes bearing covalently linked Cys-Dec-CONH_2_ (Cys-Dec-CONH_2_@Lipossomes) were prepared according to the second strategy only.

The modification of PLGA-PEG-Mal with Cys-Dec-CONH_2_ was conducted by dissolving 15 mg of the co-polymer, 2.9 mg of the peptide, and 1.9 mg of TCEP in two milliliters of anhydrous DMF and incubating the mixture under stirring (200 rpm) for 24 h at 4 °C in a nitrogen atmosphere [35]. The functionalized copolymer was then diluted with 40 mL of ultrapure water, transferred to a dialysis membrane (Pierce SnakeSkin, MWCO 3.5–10 kDa, ThermoFisher Scientific, Waltham, MA, USA) and dialyzed against water overnight. Finally, PLGA-PEG-Cys-Dec-CONH_2_ was lyophilized and stored at −20 °C under a nitrogen atmosphere. Conjugation was confirmed by proton nuclear magnetic resonance spectroscopy (Supplementary Materials), and Cys-Dec-CONH_2_@PLGA-PEG NPs [Pre-funct] were further prepared as described for PLGA NPs by replacing 4 mg of PLGA by PLGA-PEG-Cys-Dec-CONH_2_.

Cys-Dec-CONH_2_@PLGA-PEG NPs [Post-funct] were prepared by adding 0.5 mg of TCEP and 0.9 mg of the peptide (pre-mixed for 1 h at room temperature in 1 mL of 1.1 M MOPS buffer, pH 7) to a freshly prepared batch of PLGA-PEG-Mal NPs before washing (20 mg in 10 mL of 1% (*w*/*v*) poloxamer 407). The dispersion was left to react for 24 h at 4 °C under magnetic stirring (200 rpm). Functionalized NPs were then washed twice with ultrapure water and concentrated as described above. A similar procedure was used to modify liposomes with Cys-Dec-CONH_2_ (Cys-Dec-CONH_2_@Lipossomes). In this case, a freshly prepared batch of liposomes was diluted in 9 mL of 0.1 M MOPS buffer, pH 7 and further processed as described for PLGA-PEG-Mal NPs, except for the final washing step, which was performed as stated for plain liposomes.

Cys-Dec-CONH_2_ was also physically loaded into PLGA-PEG NPs (Cys-Dec-CONH_2_@PLGA-PEG NPs [Loaded]). Briefly, 16 mg of PLGA and 4 mg of PLGA-PEG were dissolved in 1 mL of acetone and mixed with 1 mL of DMF containing 1 mg of Cys-Dec-CONH_2_. The mixture was then used for producing NPs by nanoprecipitation as described above.

### 2.4. Physicochemical Characterization of Nanosystems

Average diameter and polydispersity index (PdI) were measured by dynamic light scattering (DLS) and zeta potential was determined by laser Doppler anemometry for all nanosystems using a Zetasizer Nano ZS (Malvern Panalytical, Malvern, UK). Measurements were performed at 20 °C after dilution of samples (1:100, *v*/*v*) in 10 mM sodium chloride aqueous solution.

The morphology of nanosystems was analyzed by transmission electron microscopy (TEM) using a JEOL JEM 1400 microscope (JEOL, Tokyo, Japan) at an acceleration voltage of 80 kV. Each colloidal dispersion (10 µL) was placed under a 300-mesh nickel grid, and excess liquid was removed. Staining with 1% uranyl acetate solution was also performed in the case of polymeric NPs and liposomes in order to increase image contrast.

The conjugation efficiency (CE%) of Cys-Dec-CONH_2_ to polymeric NPs was determined using the fluorescamine assay [29]. Briefly, a weighted sample of functionalized NPs or the co-polymer was dissolved in 150 µL of DMSO, mixed with 50 µL of fluorescamine solution in DMSO (0.3 mg/mL), and incubated for 15 min at room temperature. Fluorescence was then measured at 400/460 nm using a Synergy MX microplate reader (BioTek Instruments, Winooski, VT, USA), and recovered peptide determined from a calibration curve (5 µg/mL to 100 µg/mL of Cys-Dec-CONH_2_ dissolved in DMSO containing matching non-functionalized NPs/copolymer). CE% was calculated as the percentage of recovered peptide from the amount added during the conjugation process. In the case of Cys-Dec-CONH_2_@Lipossomes, CE% was determined indirectly (i.e., by assaying the amount of non-conjugated Cys-Dec-CONH_2_ from washing residues) using the Ellman’s assay [36]. Samples were diluted in 2.5 mL of reaction buffer (0.1 M sodium phosphate, pH 8.0, containing 1 mM EDTA) and mixed with 50 µL of 5,5-dithio-bis-(2-nitrobenzoic acid) (4 mg/mL) for 15 min. The absorbance was then measured at 412 nm, and the CE% was calculated similarly to polymeric NPs. Drug loading (DL%) of Cys-Dec-CONH_2_@PLGA-PEG NPs [Loaded] was also determined using the fluorescamine assay. DL% was calculated as the mass percentage of peptide relative to the total weight of NPs.

### 2.5. Antifungal Assays

The anti-*Candida* activity of peptides, nanosystems, and azoles was evaluated according to the CLSI M27-A4 microdilution broth reference protocol [37], as previously detailed [38,39]. Reference strains of *Candida* spp. used in this work were acquired from the American Type Culture Collection (ATCC, Manassas, VA, USA), and included *C. albicans* ATCC 90028, *C. albicans* ATCC 64550, *C. krusei* ATCC 6258, *C. glabrata* ATCC 2001, and *C. tropicalis* ATCC 750. These were maintained in SDA at 35 °C, 95% RH, and 5% CO_2_. Fresh cultures were prepared 24 h before antifungal experiments.

For the M27-A4 protocol, *Candida* spp. were dispersed in RPMI 1640 medium supplemented with 165 mM of MOPS (pH 7.0), plated in 96-well plates at a final concentration of 500 to 2500 cells/mL, and incubated with peptides, nanosystems, or azoles at 2-fold serial dilutions for 48 h at 35 °C, 95% RH, and 5% CO_2_. A positive control (RPMI 1640 + MOPS medium with cells) and a negative control (RPMI 1640 + MOPS medium without cells) were also included under the same conditions. The minimum inhibitory concentration (MIC) was then determined as the lowest concentration value of the interval at which no fungal growth was detected under microscopic observation. The minimum fungicidal concentration (MFC) was also assessed after plating 10 μL of the cell suspension from each well with no apparent fungal growth on SDA, and additional incubation for 24 h at 35 °C, 95% RH, and 5% CO_2_. The lowest concentration value of the interval at which no fungal growth was observed macroscopically was considered as the MFC value.

### 2.6. Cell Viability

The in vitro toxicity of Cys-Dec-CONH_2_ and Cys-Dec-CONH_2_@PLGA-PEG NPs [Post-funct] to HEC-1-A human endometrial cells (ATCC HTB-112, Manassas, VA, USA) was determined using the resazurin reduction assay [40]. Cells were maintained in McCoy’s 5A medium supplemented with 10% (*v*/*v*) heat-inactivated fetal bovine serum, 100 U/mL penicillin, and 100 μg/mL streptomycin at 37 °C, 95% RH, and 5% CO_2_. Culture medium was refreshed every 2 to 3 days, and cells reaching 70–90% confluence were sub-cultured using 0.25% (*w*/*v*) trypsin-0.53 mM EDTA as dissociation reagent.

The resazurin reduction assay was conducted by incubating 5000 cells per well in 96-well plates for 24 h before adding Cys-Dec-CONH_2_ and Cys-Dec-CONH_2_@PLGA-PEG NPs [Post-funct] at 2-fold serial dilutions and incubating for 48 h at 37 °C, 95% RH, and 5% CO_2_. Supplemented medium only and 1% (*w*/*v*) Triton X-100 in complete medium were also tested as 100% and 0% viability controls, respectively, under the same conditions. The cells were then washed twice with phosphate buffered saline (pH 7.4), and 20% (*v*/*v*) resazurin in supplemented McCoy’s 5A medium added and incubated for 3 h. Finally, individual supernatants were transferred to a new 96-well plate, and fluorescence was measured at 530/590 nm using a Synergy Mx microplate reader. Half-maximal cytotoxic concentration (CC_50_) values were calculated by log-logistic regression of viability percentage versus concentration plots using Prism 9 (GraphPad, San Diego, CA, USA).

### 2.7. Statistical Analysis

All experiments were performed in triplicate, and results are presented as mean ± standard deviation (SD), unless otherwise stated. MIC and MFC values are shown as single value or interval of concentrations at which no fungal growth was observed. Multiple comparisons were performed by one-way ANOVA with Tukey’s post hoc test using Prism (v. 8, GraphPad Software, La Jolla, CA, USA). Values of *p* < 0.05 were considered as denoting significance.

## 3. Results and Discussion

### 3.1. Colloidal Properties of Nanosystems

**Figure 2:**
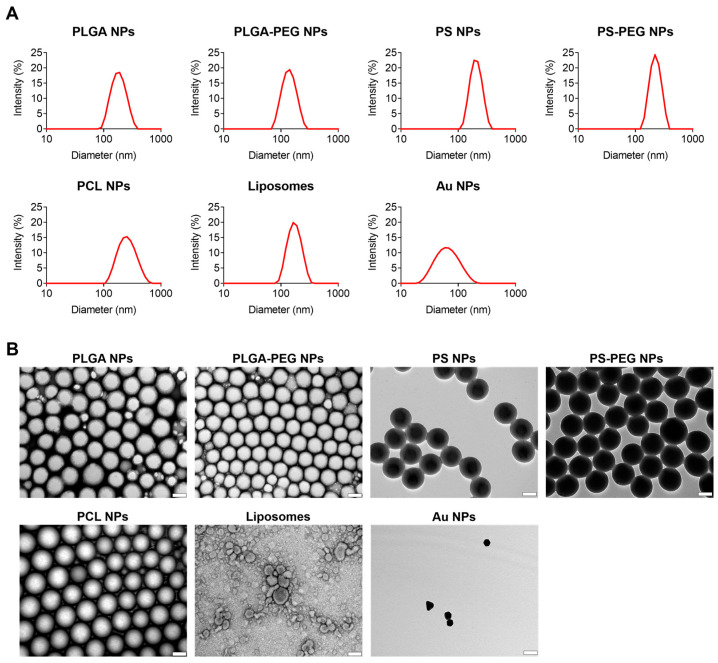
Size distribution and morphology of tested nanosystems. (**A**) Representative size distribution plots as determined by DLS; and (**B**) TEM images (scale bars = 100 nm).

We obtained different types of NPs, either in-house or from commercial sources (PS NPs and Au NPs). Densely PEGylated PLGA and PS NPs were prepared by using copolymers (PLGA-PEG) or by modifying pre-prepared PS NPs via carbodiimide chemistry, as described previously by our team [41]. All NPs were evaluated for hydrodynamic diameter, size distribution, and charge (Table 1). Most nanomaterials featured hydrodynamic diameter values in the range of 150–250 nm, except for Au NPs, which were considerably smaller. All nanosystems generally exhibited fairly narrow and monomodal size distributions, as inferred by their PdI values and size distribution plots (Figure 2A). The zeta potential was near neutral not only for PEG-coated systems, but also for PCL NPs (Table 1). In this last case, the simple use of poloxamer 407 as stabilizer was able to shield the typically negatively charged surface of particles [42]. PLGA NPs showed mildly negative zeta potential, indicating a lesser shielding effect of poloxamer 407 in this case. Conversely, PS NPs and Au NPs featured markedly negative zeta potential, which is expected due to the presence of carboxylate groups in the polymeric system and the presence of citrate used as a stabilizer in the metallic particles. Such differences could be potentially relevant, as previous work showed that surface charge may be important in defining the adhesion potential of NPs to *C. albicans* [43].

The morphology of nanosystems was further assessed by TEM imaging (Figure 2B). All polymeric NPs featured spherical shape and size ranges in line with those determined by DLS. Liposomes were generally roundish but slightly deformed in some cases, highlighting the typical flexibility of such vesicles. The presence of a single lipid bilayer could also be inferred, reinforcing their unilamellar structure. As for Au NPs, images showed nanosystems with well-defined but irregular shapes, presumably due to their crystalline organization. The diameter values determined by DLS for these metallic NPs were also confirmed.

### 3.2. Antifungal Activity of Dec-CONH_2_ and Cysteine-Modified Derivatives

The anti-*Candida* activity of Dec-CONH_2_ has been previously described [21,22], but the effects of adding a cysteine residue have not been assessed. We started by testing the antifungal activity of cysteine-modified Dec-CONH_2_ against a panel of reference strains of *Candida* spp. and compared it to the non-modified peptide (Table 2). Both *N*-terminal (Cys-Dec-CONH_2_) and *C*-terminal (Dec-CONH_2_-Cys) modified peptides were tested. Fluconazole and clotrimazole were also included for reference to azole resistance of the different strains. The values of MIC and MFC for these two azoles against susceptible *Candida* spp. strains agreed with their known potency and data obtained using the CLSI M27 reference protocol [44,45,46,47]. Values of MIC and MFC for Dec-CONH_2_ against *C. albicans* were also in line with reported IC_50_ values (~9–27 µg/mL) [21,22]. The addition of cysteine on the *N*-terminal did not change the activity of Dec-CONH_2_, with values remaining within one log2 range [37]. However, changes to the amidated terminal (*C*-terminal) typically led to a marked reduction in activity (four-fold increase or higher in MIC or MFC). Dec-CONH_2_ and its derivatives did not seem to feature reduced activity against azole-resistant *albicans* (ATCC 64550) and non-*albicans* (*C. krusei ATCC 6258*) strains.

### 3.3. Antifungal Activity of Cys-Dec-CONH_2_ in the Presence of Nanosystems

For the purpose of screening the activity of Cys-Dec-CONH_2_ in the presence of nanosystems, we selected two strains: *C. albicans* ATCC 90028 representing more prevalent, azole-sensitive organisms, and *C. krusei* ATCC 6258 as illustrative of more virulent and intrinsically azole-resistant non-*albicans* yeasts [6,48]. All bare NPs were firstly tested as controls in order to discard the possibility of any intrinsic anti-*Candida* activity. For instance, some studies reported that Au NPs may possess antifungal activity [49,50,51]. However, none of the nanosystems considered in the present work featured intrinsic activity against either *C. albicans* or *C. krusei* (MIC and MFC values above concentrations of nanosystems equivalent to >256 µg/mL of Cys-Dec-CONH_2_ in ratios considered for physical mixtures). The absence of activity for Au NPs for the NPs used in our work as compared to other reports may be explained by different colloidal properties (size, shape, or surface chemistry) or simply by the use of a more sensitive, clinically relevant antifungal testing protocols such as CLSI M27-A4.

We continued the study by assessing the anti-*Candida* activity of physical mixtures of Cys-Dec-CONH_2_ with different nanosystems (Table 3), before considering any association between peptide and nanosystems. Changes in activity were noticeable, particularly in the case of PLGA NPs, PLGA-PEG NPs, and PCL NPs. The simple presence of such nanosystems rendered the peptide inactive against *Candida*, as noted before for other peptide–nanosystem conjugates [25]. Such effect was even observed when the fungus was left in contact with the peptide for up to 36 h, and PLGA NPs or PLGA-PEG NPs were added only during the last 12 h of the incubation period. These data suggest that Cys-Dec-CONH_2_ requires considerable contact time with *Candida* in order to fully exert its antifungal action, but its inactivation by nanosystems occurs rapidly and extensively, as similarly reported for other antimicrobial peptides adsorbed onto various materials [52,53]. One common feature of PLGA NPs, PLGA-PEG NPs, and PCL NPs is the use of poloxamer 407 during production. Even if this particle stabilizer is extensively removed during washing steps, it can still adsorb in substantial amounts at the surface of NPs [42,54]. Such an effect was evidenced by the near-neutral zeta potential values obtained for most NPs. In order to discard the possibility that residual poloxamer 407 interferes with the anti-*Candida* activity, we tested the ability of Cys-Dec-CONH_2_ in the presence of 1% (concentration used during production) and 5% of the particle stabilizer. While the highest concentration inhibited the activity of the peptide (MIC and MFC values over 256 µg/mL), the lowest level resulted in no substantial changes (MIC and MFC values of 64 µg/mL for *C. albicans* ATCC 90028 and 16 µg/mL for *C. krusei* ATCC 6258). These results suggest that poloxamer 407 was not implicated in the decrease in peptide activity when mixed with PLGA NPs, PLGA-PEG NPs, and PCL NPs.

Mixtures of the peptide with other nanosystems appeared to have little to no effect (Table 3), with only negligible differences (MIC and MFC values within one log2 range) being observed for PS NPs, PS-PEG NPs, Au NPs, and liposomes in the case of *C. albicans*. Results for *C. krusei* were similar, although a mild reduction in activity was observed for PS NPs and liposomes (MIC and MFC values were four-fold higher). Overall, it seems that Cys-Dec-CONH_2_ has a high tendency to interact with polymeric NPs, which could reduce the availability of the peptide to interact with fungi [24]. PS NPs were the most hydrophobic type of polymeric nanosystem tested in this study [55,56], and this fact alone may have contributed to less affinity of hydrophilic Cys-Dec-CONH_2_ to adsorb at the surface of the PS NPs, thus leading to only a minor effect on activity. The explanation seems to be less clear in the case of liposomes, but the amphiphilic nature of these vesicles may contribute to only mild adsorption of Cys-Dec-CONH_2_ at their surface [57,58].

The anti-*Candida* activity data suggest that different nanosystems are able to interact to a different extent with Cys-Dec-CONH_2_. In order to further discard if any changes were induced to the native structure of the peptide, we incubated it overnight with PLGA-PEG NPs and tested the activity after recovery. Results were similar to those for native Cys-Dec-CONH_2_ (MIC = 64 µg/mL and MFC = 128 µg/mL for *C. albicans*; MIC = 32 µg/mL and MFC = 32 µg/mL for *C. krusei*), thus suggesting that the peptide structure was fairly maintained. This points to an alternative mechanism of interference related with interfacial interactions between the peptide and nanosystems. The cationic nature of Cys-Dec-CONH_2_ is likely to promote its tight adsorption onto mildly negatively charged PLGA-PEG NPs [59,60].

### 3.4. Antifungal Activity of Nanosystems Incorporating Cys-Dec-CONH_2_

We wanted to understand if the puzzling results obtained for the physical mixtures could be reversed by associating the peptide with NPs in a more controlled fashion, or if the close proximity with nanosystems could in fact inactivate Cys-Dec-CONH_2_. For example, previous work by Pal et al. showed that covalent conjugation of odorranain-A-OA1, an antimicrobial cationic peptide, to silver NPs was able to enhance activity against the bacterium *E. coli*, contrasting with the inhibition of the peptide when simply mixed with NPs [24]. This effect was explained by the strong electrostatic interaction between peptide and NPs that rendered odorranain-A-OA1 partially inactivated. We selected PLGA-PEG-based carriers for further experiments due to their biodegradability and PEG shielding, which is able to provide prolonged blood circulation upon intravenous injection [61,62] and mucus-diffusive properties when administered at mucosal sites [63,64]. The covalent bonding of Cys-Dec-CONH_2_ to the co-polymer was performed before (Cys-Dec-CONH_2_@PLGA-PEG NPs [Pre-funct]) or after (Cys-Dec-CONH_2_@PLGA-PEG NPs [Post-funct]) producing NPs. The cysteine residue of the peptide was used for site-oriented covalent bonding to the copolymer featuring a terminal maleimide group at the PEG arm. Both types of functionalized NPs presented colloidal properties similar to plain PLGA-PEG NPs (Table 4). We also considered the physical entrapment of the peptide in the polymeric matrix of NPs, as well as surface functionalized liposomes with the peptide for comparison purposes. In all cases, the cationic nature of the peptide was not able to substantially change the zeta potential of the nanosystems.

Results from anti-*Candida* activity showed that, irrespective of the strategy used, all nanosystems rendered Cys-Dec-CONH_2_ inactive (Table 5). This was particularly striking for liposomes. Although the physical mixture resulted only in partial impairment of activity (Table 3), surface immobilization of the peptide rendered its complete inactivation, suggesting that the close proximity of nanosystems and the peptide is able to interfere with its ability to interact with the fungus. In the case of PLGA-PEG NPs, neither oriented surface functionalization (which promoted maximum exposure of the amidated *C*-terminal of the peptide) nor non-covalent encapsulation (which could help protect the peptide and promote sustained release) were able to counteract interference with antifungal activity. Similar results were reported by Wu et al. for cecropin P1 covalently immobilized onto PEGylated silica NPs when tested for antimicrobial activity against *E. coli* [65]. Ramôa et al. also noted that immobilizing the MSI-78(4–20) peptide onto PLGA-PEG NPs led to a reduction in the activity against bacteria such as *S. aureus* and *P. aeruginosa* [29]. Such an effect seems to be particularly common and challenging when considering antimicrobial peptide immobilization onto coatings of medical devices and implants [66,67].

**Figure 3:**
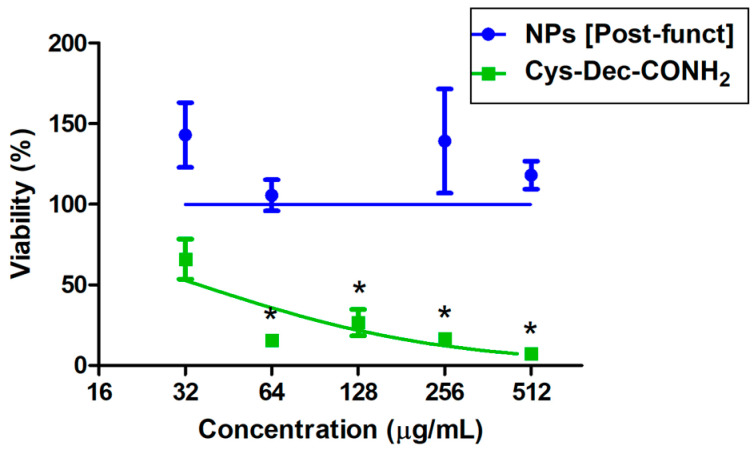
Viability of HEC-1-A human endometrial cells in the presence of Cys-Dec-CONH_2_ or Cys-Dec-CONH_2_@PLGA-PEG NPs [Post-funct]. Results presented as mean ± standard error of the mean (concentrations expressed as total Cys-Dec-CONH_2_; *n* = 3). Lines represent log-logistic regression plots of data. (*) Indicates significant differences (*p* < 0.05) as compared to negative control (100% viability).

The cytotoxicit0079 of Cys-Dec-CONH_2_ to HEC-1-A human endometrial cells was also studied, in order to understand if the interaction with nanosystems was also able to inhibit other biological effects of the peptide, as observed in various other studies [25]. For that, we tested Cys-Dec-CONH_2_@PLGA-PEG NPs [Post-funct], and results (Figure 3) showed a substantial shift in the toxicity concentration threshold (CC_50_ > 512 µg/mL in total peptide) as compared to the peptide alone (CC_50_ = 36 µg/mL). These data appear to confirm that surface immobilization of Cys-Dec-CONH_2_ at the surface of PLGA-PEG NPs is able to inhibit the establishment of interactions between peptide and HEC-1-A human cells.

### 3.5. Antifungal Activity of Cys-Dec-CONH_2_ in the Presence of Biomolecules

The data presented above suggest that the antifungal activity of Cys-Dec-CONH_2_ can easily be partially or completely disrupted, likely by direct interfacial interaction with nanosystems. Thus, we pondered whether this inactivation could also occur in the presence of biologically relevant macromolecules. We mixed Cys-Dec-CONH_2_ with either mucin or albumin at different concentrations and tested their anti-*Candida* activity (Table 6). Again, interference was observed for both macromolecules. Mucin is the main component of fluids present at mucosal sites, with concentrations ranging from as little as 0.01% in the eye to nearly 5% in the stomach [68,69,70]. While reduction of the antifungal activity of Cys-Dec-CONH_2_ was negligible at 0.1% mucin, extensive inhibition was noted at 1% mucin. Felgentreff et al. [71] and Huang et al. [72] found similar consequences for the antibacterial activity of polymyxins and cathelicidin, respectively, when mixed with mucin, thus suggesting that such effect should be screened early in the development of new antimicrobial peptides. Albumin was chosen as the most abundant protein in the blood, proxying the scenario of systemic distribution of Cys-Dec-CONH_2_. Reduction of the anti-*Candida* activity was observed even for concentrations much lower than the normal serum levels (3.5–5%) [73]. Similarly, Tang et al. observed that fetal bovine albumin was able to inhibit the activity of the peptide TP4 against *C. albicans* due to complexation between the two molecules [74].

## 4. Conclusions

Antifungal peptides and their association with nanocarriers offer great promise for advancing antifungal therapeutics, but their translation into tangible products remains challenging. In this work, we provide evidence that the mere presence of various nanosystems can impair, to different degrees, the ability of Cys-Dec-CONH_2_ to inhibit *Candida* spp. using a clinically relevant assay. The effect was particularly striking for PLGA NPs and PLGA-PEG NPs. Although the addition of the cysteine residue to the *N*-terminal of Dec-CONH_2_ did not substantially affect antifungal activity, functionalization of different nanosystems with Cys-Dec-CONH_2_ via covalent bonding or encapsulation led to complete loss of antifungal activity. This effect seems to be correlated with the strong interfacial electrostatic interactions between peptide and nanomaterial, as similarly described for antibacterial peptides. Additionally, two biorelevant macromolecules—albumin and mucin—were also able to inhibit the anti-*Candida* activity of Cys-Dec-CONH_2_ at levels close to those observed in biological fluids. This supports the need to test antifungal peptides under conditions mimicking biological settings relevant to their prospective clinical applications.

## Figures and Tables

**Table 1:** Colloidal properties of nanosystems. Results are presented as mean ± SD (*n* = 3).

Nanosystem	Diameter (nm)	PdI	Zeta Potential (mV)
PLGA NPs	174 ± 4	0.093 ± 0.021	−11.7 ± 1.3
PLGA-PEG NPs	142 ± 12	0.090 ± 0.010	−7.6 ± 1.2
PCL NPs	247 ± 6	0.147 ± 0.016	−3.2 ± 0.5
PS NPs	200 ± 2	0.025 ± 0.014	−64.3 ± 2.0
PS-PEG NPs	218 ± 3	0.024 ± 0.013	−2.3 ± 2.3
Au NPs	53 ± 0	0.249 ± 0.007	−32.4 ± 1.5
Liposomes	168 ± 7	0.118 ± 0.051	−3.9 ± 1.2

**Table 2:** Antifungal activity of Dec-CONH_2_, Cys-Dec-CONH_2_, Dec-CONH_2_-Cys, and two azoles. Results are presented as MIC and MFC (values expressed in µg/mL; *n* = 3).

	*C. albicans* ATCC 90028		*C. albicans* ATCC 64550		*C. krusei* ATCC 6258		*C. glabrata* ATCC 2001		*C. tropicalis* ATCC 750	
	MIC	MFC	MIC	MFC	MIC	MFC	MIC	MFC	MIC	MFC
Dec-CONH_2_	32	32	32	32	32	32	64	64	8	16
Cys-Dec-CONH_2_	64	64	64	64	16	16	32	32	16	16
Dec-CONH_2_-Cys	128	128	128	128	64	64	128	256	16–32	32
Fluconazole	0.5–1	>256	>256	>256	64	128	16–32	>256	2	>256
Clotrimazole	1	2	4	>256	<1	1	2	8–16	4	16

**Table 3:** Antifungal activity of Cys-Dec-CONH_2_ in the presence of different nanosystems (physical mixture). Results are presented as MIC and MFC (values expressed in µg/mL of Cys-Dec-CONH_2_; *n* = 3). Values for plain Cys-Dec-CONH_2_ are also included for reference.

	*C. albicans* ATCC 90028		*C. krusei* ATCC 6258	
	MIC	MFC	MIC	MFC
Cys-Dec-CONH_2_	64	64	16	16
Cys-Dec-CONH_2_ + PLGA NPs	>256	>256	>256	>256
Cys-Dec-CONH_2_ + PLGA-PEG NPs	>256	>256	>256	>256
Cys-Dec-CONH_2_ + PLGA NPs (12 h) ^(a)^	>256	>256	>256	>256
Cys-Dec-CONH_2_ + PLGA-PEG NPs (12 h) ^(a)^	>256	>256	>256	>256
Cys-Dec-CONH_2_ + PCL NPs	>256	>256	>256	>256
Cys-Dec-CONH_2_ + PS NPs	64	64	64	64
Cys-Dec-CONH_2_ + PS-PEG NPs	64	64	32	32
Cys-Dec-CONH_2_ + Au NPs	128	128	32	32
Cys-Dec-CONH_2_ + liposomes	128	128	64	64

^(a)^ NPs were only added during the last 12 h of the 48 h incubation period.

**Table 4:** Colloidal properties of nanosystems modified with Cys-Dec-CONH_2_. Results are presented as mean ± SD (*n* = 3).

	Diameter (nm)	PdI	Zeta Potential (mV)	CE%	DL%
Cys-Dec-CONH_2_@PLGA-PEG NPs [Pre-funct]	162 ± 5	0.075 ± 0.010	−3.0 ± 1.7	30 ± 12	4.1 ± 3.3
Cys-Dec-CONH_2_@PLGA-PEG NPs [Post-funct]	143 ± 6	0.105 ± 0.028	−1.6 ± 0.9	33 ± 20	2.6 ± 1.6
Cys-Dec-CONH_2_@PLGA-PEG NPs [Loaded]	168 ± 1	0.097 ± 0.029	−4.7 ± 0.6	N.A.	1.1 ± 0.1
Cys-Dec-CONH_2_@Lipossomes	169 ± 3	0.128 ± 0.023	−2.5 ± 1.3	94 ± 0	2.2 ± 0.0

N.A.: not applicable.

**Table 5:** Antifungal activity of selected nanosystems after incorporation of Cys-Dec-CONH_2_. Results are presented as MIC and MFC (values expressed in µg/mL of Cys-Dec-CONH_2_; *n* = 3). Values for plain Cys-Dec-CONH_2_ are also included for reference.

	*C. albicans* ATCC 90028		*C. krusei* ATCC 6258	
	MIC	MFC	MIC	MFC
Cys-Dec-CONH_2_	64	64	16	16
Cys-Dec-CONH_2_@PLGA-PEG NPs [Pre-funct]	>256	>256	>256	>256
Cys-Dec-CONH_2_@PLGA-PEG NPs [Post-funct]	>256	>256	>256	>256
Cys-Dec-CONH_2_@PLGA-PEG NPs [Loaded]	>256	>256	>256	>256
Cys-Dec-CONH_2_@Lipossomes	>256	>256	>256	>256

**Table 6:** Antifungal activity of Cys-Dec-CONH_2_ in the presence of mucin or albumin. Results are presented as MIC and MFC (values expressed in µg/mL of Cys-Dec-CONH_2_; *n* = 3). Values for plain Cys-Dec-CONH_2_ are also included for reference.

	*C. albicans* ATCC 90028		*C. krusei* ATCC 6258	
	MIC	MFC	MIC	MFC
Cys-Dec-CONH_2_	64	64	16	16
Cys-Dec-CONH_2_ + mucin (0.1%)	64	64	32	32
Cys-Dec-CONH_2_ + mucin (1%)	>256	>256	>256	>256
Cys-Dec-CONH_2_ + albumin (0.1%)	128	128	32	32
Cys-Dec-CONH_2_ + albumin (1%)	>256	>256	>256	>256

## Data Availability

The data presented in this study are available from the corresponding author upon reasonable request.

## Supplementary Materials

The following supporting information can be downloaded at: https://www.mdpi.com/article/10.3390/pharmaceutics17040460/s1, Characterization of PLGA-PEG-Cys-Dec-CONH_2_ by Nuclear Magnetic Resonance Spectroscopy; Figure S1: ^1^H NMR spectra for Cys-Dec-CONH_2_, PLGA-PEG-Cys-Dec-CONH_2_, and PLGA-PEG-Mal. Reference [75] is cited in the Supplementary Materials.

## Abbreviations

The following abbreviations are used in this manuscript:

Au NPs	Gold nanoparticles
CC_50_	Half-maximal cytotoxic concentration
CE%	Conjugation efficiency percentage
Cys-Dec-CONH_2_	*N*-terminal cysteine-modified Dec-CONH_2_
Cys-Dec-CONH_2_@Lipossomes	Liposomes post-functionalized with Cys-Dec-CONH_2_
Cys-Dec-CONH_2_@PLGA-PEG NPs [Loaded]	Cys-Dec-CONH_2_-loaded PLGA-PEG NPs
Cys-Dec-CONH_2_@PLGA-PEG NPs [Post-funct]	PLGA-PEG NPs post-functionalized with Cys-Dec-CONH_2_
Cys-Dec-CONH_2_@PLGA-PEG NPs [Pre-funct]	PLGA-PEG NPs pre-functionalized with Cys-Dec-CONH_2_
Dec	Decoralin
Dec-CONH_2_	*C*-terminal amidated decoralin
Dec-CONH_2_-Cys	*C*-terminal cysteine-modified Dec-CONH_2_
DL%	Drug loading percentage
MFC	Minimum fungicidal concentration
MIC	Minimum inhibitory concentration
PCL NPs	Polycaprolactone nanoparticles
PdI	Polydispersity index
PLGA NPs	Poly(d,l-lactic-co-glycolic acid) nanoparticles
PLGA-PEG NPs	Poly(d,l-lactic-co-glycolic acid)-poly(ethylene glycol) nanoparticles
PLGA-PEG-Mal NPs	Poly(d,l-lactic-co-glycolic acid)-poly(ethylene glycol)-maleimide
PS NPs	Polystyrene nanoparticles
PS-PEG NPs	Polystyrene-poly(ethylene glycol) nanoparticles
