# Supplementary material for: Anti-Candida Activity of Cysteine-Modified Amidated Decoralin in the Presence of Engineered Nanomaterials
Source: Pharmaceutics. 2025 Apr 2;17(4):460. doi: 10.3390/pharmaceutics17040460 (PMC12030351; doi:10.3390/pharmaceutics17040460)
Supplement: Supplementary file 1 [file pharmaceutics-17-00460-s001.zip › pharmaceutics-3549592-supplementary.pdf]

## Supplementary Materials

Characterization of PLGA-PEG-Cys-Dec-$CONH_2$ by Nuclear Magnetic Resonance Spectroscopy

Proton nuclear magnetic resonance ($^1H$ NMR) spectroscopy analysis was performed to confirm the conjugation between Cys-Dec-$CONH_2$ and PLGA-PEG-Mal. Lyophilized samples were dissolved in deuterated dimethyl sulfoxide and NMR analysis was performed using a Bruker Avance III 400 MHz spectrometer (Bruker, Billerica, MA, USA). Chemical deviations were expressed in ppm (δ units) and MestReNova software (v. 14) was used for data analysis. Spectra for Cys-Dec-$CONH_2$, PLGA-PEG-Cys-Dec-$CONH_2$ and PLGA-PEG-Mal are presented in Figure S1. Four characteristic peaks of PLGA-PEG were found in both polymers, namely at 5.2 ppm (corresponding to –CH protons in lactic acid monomers), 4.8 ppm (corresponding to –$CH_2$ protons in glycolic acid monomers), 3.6 ppm (corresponding to –$CH_2$ protons in ethylene glycol monomers) and 1.5 ppm (corresponding to –$CH_3$ protons in lactic acid monomers) [1, 2]. In the case of PLGA-PEG-Cys-Dec-$CONH_2$, the presence of two peaks (around 1.1 ppm and 2.1 ppm) suggests functionalization with the peptide.

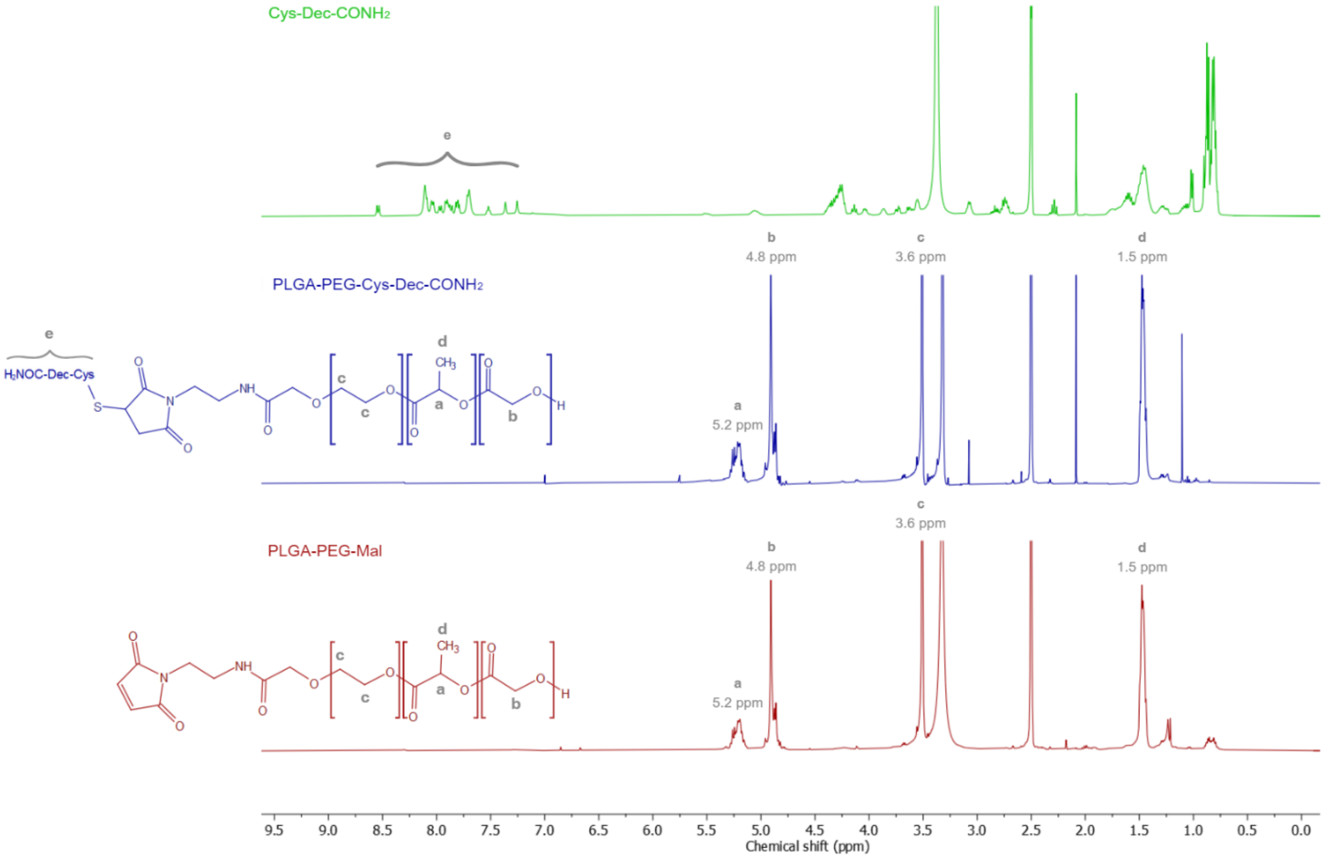


Figure S1. $^{1}H$ NMR spectra for Cys-Dec-$CONH_2$, PLGA-PEG-Cys-Dec-$CONH_2$ and PLGA-PEG-Mal.

## Supplementary references


1. Ramôa, A. M.; Campos, F.; Moreira, L.; Teixeira, C.; Leiro, V.; Gomes, P.; das Neves, J.; Martins, M. C. L.; Monteiro, C., Antimicrobial peptide-grafted PLGA-PEG nanoparticles to fight bacterial wound infections. *Biomater Sci* **2023,** *11*, 499-508.
2. Silveira, M. J.; Martins, C.; Cruz, T.; Castro, F.; Amorim-Costa, A.; Chester, K.; Oliveira, M. J.; Sarmento, B., scFv biofunctionalized nanoparticles to effective and safe targeting of CEA-expressing colorectal cancer cells. *J Nanobiotechnology* **2023,** *21*, 357.
